# The Clinical Relevance of an Inflamed Appendix in Crohn’s Disease

**DOI:** 10.1093/ecco-jcc/jjad202

**Published:** 2023-12-01

**Authors:** Eline M L van der Does de Willebois, Cagla Sari, Aart Mookhoek, Vincent Joustra, Susan van Dieren, Geert R D’Haens, Willem A Bemelman, Christianne J Buskens

**Affiliations:** Department of Surgery, Amsterdam University Medical Center, AMC, Amsterdam, The Netherlands; Department of Surgery, Amsterdam University Medical Center, AMC, Amsterdam, The Netherlands; Institute of Tissue Medicine and Pathology, University of Bern, Bern, Switzerland; Department of Gastroenterology and Hepatology, Amsterdam University Medical Center, AMC, Amsterdam, The Netherlands; Department of Surgery, Amsterdam University Medical Center, AMC, Amsterdam, The Netherlands; Department of Gastroenterology and Hepatology, Amsterdam University Medical Center, AMC, Amsterdam, The Netherlands; Department of Surgery, Amsterdam University Medical Center, AMC, Amsterdam, The Netherlands; Department of Surgery, Amsterdam University Medical Center, AMC, Amsterdam, The Netherlands

**Keywords:** Crohn’s disease, ileocolic resection, appendicular inflammation

## Abstract

**Background and Aims:**

An appendectomy for appendiceal inflammation has been suggested to ameliorate the clinical course of patients with ulcerative colitis [UC]. In contrast, for Crohn’s disease [CD] an inverse association has been suggested with a higher incidence of CD and worse prognosis after appendectomy. The aim of this study was to analyse the clinical relevance of an inflamed appendix in CD patients undergoing ileocaecal resection [ICR].

**Methods:**

All consecutive patients undergoing primary ICR between 2007 and 2018 were considered for inclusion. Microscopic data of available appendiceal resection specimens [*n* = 99] were revised by a dedicated inflammatory bowel disease [IBD] pathologist and scored as inflamed or not inflamed. Eighteen patients had a previous appendectomy. Pathological findings were correlated with disease characteristics and recurrence rates [clinical, endoscopic, and intervention-related].

**Results:**

In total 117 patients were included, 77 [65.8%] females, with a median age of 30 years (interquartile range [IQR] 24–43), and a median follow up of 102 months [IQR 76–114]. Of patients without previous appendectomy [*n* = 99], 39% had an inflamed appendix. No significant differences in disease characteristics [eg, disease location, behaviour, time to surgery] or prognosis could be demonstrated between the two groups. In contrast, previous appendectomy [*n* = 18] was associated with penetrating disease and numerically shorter disease duration at the time of resection. Furthermore, a trend was seen towards a stronger association with postoperative recurrence.

**Conclusion:**

The current study could not confirm a different prognosis for CD patients with and without an inflamed appendix. In contrast, in patients with a previous appendectomy, a trend was seen towards increased postoperative recurrence, which might be related to the higher incidence of penetrating disease.

## 1. Introduction

The appendix, a previously suggested vestigial remnant, is the pouch-like beginning of the large intestine. There is increasing evidence that the appendix has an active immunomodulatory function which includes housing and cultivating beneficial gut flora that can repopulate the digestive system.^1^ It has been demonstrated that an appendectomy during childhood decreases the risk of developing ulcerative colitis [UC], and in patients with UC, an appendectomy might decrease relapses and reduce the need for medication.^2–4^ This effect was suggested to be associated with active inflammation of the appendix, which was even seen in over 50% of UC patients both in remission and with active disease.^5^ In contrast, a systematic review on the role of the appendix in Crohn’s disease [CD] showed an inverse correlation: it was demonstrated that an appendectomy is associated with the development of CD, particularly in the first years after surgery, and that CD patients show worse prognosis after appendectomy.^6,7^ Data on the association between an inflamed appendix in the resection specimen at the time of ileocaecal resection [ICR] and its effect on CD prognosis are lacking.

Postoperative recurrences in CD are the rule rather than an exception.^8^ Several risk factors have been identified: perianal disease, penetrating disease, smoking, and prior intestinal surgery.^9^ Recurrences are also associated with disease location, with less than 20% re-resections after 10 years in disease located in the terminal ileum [L1 disease], whereas the prognosis of patients with concomitant colonic disease [L2 and L3 disease] is worse.^10^ In L3 patients, up to 88% develop local recurrence, and up to 75% of L2 patients require reoperation after colonic resection.^11–13^

As the appendix is anatomically part of the colon, the hypothesis of the current study is that patients with signs of an inflamed appendix are more likely to have colonic disease rather than pure small bowel disease, with an associated worse postoperative prognosis. This could result in the identification of a patient group more likely to benefit from postoperative prophylactic medication.

The aim of this study was to analyse the incidence of inflamed appendices in patients undergoing ICR for CDm and to correlate findings with disease characteristics and postoperative prognosis. In addition, results will be compared with the prognosis of patients who previously underwent appendectomy.

## 2. Materials and Methods

### 2.1. Design and patients

Data of all consecutive patients undergoing resection for CD in the Amsterdam UMC are collected in a prospectively maintained database. Patients aged ≥18 years with terminal ileitis [L1 and L3 disease], who underwent primary ileocaecal resection between 2007 and 2018, were included in the current retrospective study. Patients were excluded if they had less than 3 years follow-up, were demonstrated to have a malignancy in the resection specimen, were lacking microscopic histological data on the appendix, or objected to the use of data. Reporting of the data adheres to the STROBE Statement.^14^

### 2.2. Histological features

Pathology reports were reviewed by a dedicated gastrointestinal pathologist specialised in inflammatory bowel disease [IBD]. Patients were divided into three groups based on appendicular [histological] data: inflamed appendix at the time of ICR, not inflamed [no signs of inflammation] appendix at the time of ICR, or previous appendectomy. An inflamed appendix was defined as follows: signs of neutrophilic granulocytes, crypt abscesses, erosion and ulceration, and/or chronic inflammation. In case of total fibrous obliteration of the lumen, the appendix was considered not inflamed. Previous appendectomy was defined as appendectomy prior to ICR.

### 2.3. Variables and outcomes

The primary endpoints were the incidence of inflamed appendices in patients undergoing primary ICR for CD and its correlation with disease characteristics and prognosis [recurrence rates]. In addition, the results of patients with and without an inflamed appendix in the resection specimen were compared with outcomes in patients who previously underwent appendectomy.

The prognosis was expressed as the development of postoperative [endoscopic and/or clinical] recurrence and intervention-related recurrence. Postoperative endoscopic recurrence was defined as a modified Rutgeerts score of ≥i2b. Clinical recurrence was defined as the development of Crohn’s-related symptoms [diarrhoea, fever, rectal bleeding, abdominal pain, or evident fatigue] objectively confirmed by elevated biomarker levels (C-reactive protein [CRP] ≥50 mg/L or faecal calprotectin ≥250 μg/g], resulting in [re-]starting, switching, or intensifying medical therapy, preferably confirmed by colonoscopy. Intervention-related recurrence was defined as the need for re-resection for recurrent ileal CD, and balloon dilatation in case of a Rutgeerts score of i4 or stricturoplasty.

### 2.4. Statistical analysis

Differences in baseline characteristics between patients with and without an inflamed appendix and previous appendectomy, were assessed using a chi square test for categorical variables, or in case of low counts [<5], a Fisher’s exact test. The Mann–Whitney U or the Kruskal–Wallis test was used for numerical variables, as appropriate. All data were reported as median with interquartile range [IQR], mean with standard deviation [SD], or percentages [%] of the total cohort when appropriate. Kaplan–Meier analysis with log rank test was used to compare [endoscopic and/or clinical] recurrence-free survival between the group with previous appendectomy and the groups with and without an inflamed appendix at time of ICR. Two-sided *p*-values of less than 0.05 were considered statistically significant; *p*-values and confidence intervals [CIs] were calculated at a 95% confidence level. For statistical analyses, SPSS Statistics version 28 [IBM] was used.

### 2.5. Ethical considerations

This study was waived from review of the medical ethics board.

## 3. Results

**Figure 1:**
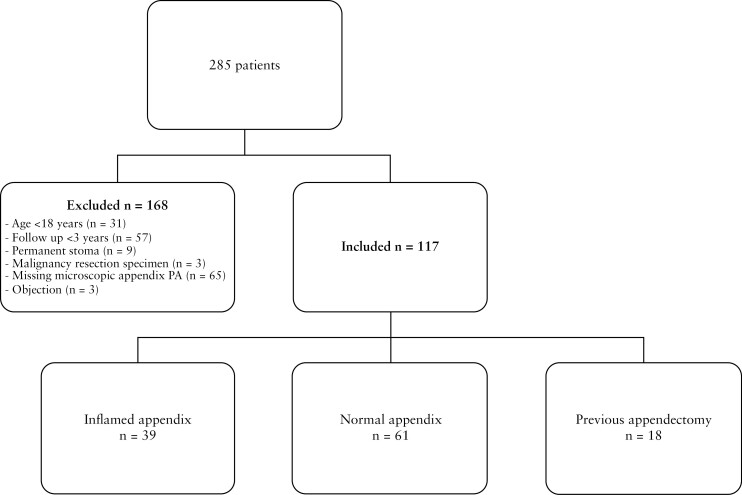
Flowchart.

A total of 285 patients was identified from the prospectively maintained database, of whom 117 patients were included in this study [Figure 1]. The median age of the total cohort was 30 years [IQR 24–43], with 77 [65.8%] females, and a median disease duration of 44 months [IQR 12–114] at the time of ICR. A total of 18 patients [9.9%] had previously undergone appendectomy, with a median time between appendectomy and ICR of 13 years [IQR 2.0–26.5]. The majority of patients underwent appendectomy prior to CD diagnosis [75%]. Baseline characteristics of the three groups are shown in Table 1.

**Table 1:** Baseline patients and disease characteristics, inflamed appendix vs not inflamed appendix vs previous appendectomy.

Baseline characteristics, *n* [%]					
	Total [*n* = 117]	Inflamed appendix [*n *= 38]	Not inflamed appendix [*n* = 61]	Appendectomy [*n* = 18]	*p*-value
Gender, female	77 [65.8]	25 [65.8]	43 [70.5]	9 [50]	0.296
Age at surgery [years], median IQR	30 [24–43]	27 [22–37]	31 [25–44]	35 [25–48]	0.104
Duration of disease [months], median IQR	44 [12–114]	41 [8–72]	49 [18–127]	37 [8–79]	0.228
Smoking, active	34 [29.1]	12 [31.6]	21 [34.4]	1 [5.6]	0.044
Age at diagnosis					0.733
Montreal A1, <17 years	13 [11.1]	6 [15.8]	5 [8.2]	2 [11.1]	
Montreal A2, 17–40 years	87 [74.4]	28 [73.7]	46 [75.4]	13 [72.2]	
Montreal A3, >40 years	17 [14.5]	4 [10.5]	10 [16.4]	3 [16.7]	
Location of disease					0.785
Montreal L1, terminal ileitis	72 [61.5]	23 [60.5]	39 [63.9]	10 [55.6]	
Montreal L3, ileocolic	45 [38.5]	15 [39.5]	22 [36.1]	8 [44.4]	
Behaviour of disease at surgery					0.045
Montreal B1, inflammatory	29 [24.8]	14 [36.8]	12 [19.7]	3 [16.7]	0.068
Montreal B2, stricturing	44 [37.6]	9 [23.7]	30 [49.2]	5 [27.8]	0.031
Montreal B3, penetrating	44 [37.6]	15 [39.5]	19 [31.1]	10 [55.6]	0.072
Perianal disease	24 [20.5]	10 [26.3]	11 [18]	3 [16.7]	0.578
Preoperative therapy <12 weeks					
Steroids	49 [41.9]	15 [39.5]	27 [44.3]	7 [38.9]	0.873
Thiopurines	45 [38.5]	17 [44.7]	21 [34.4]	7 [38.9]	0.594
Biologics	40 [34.2]	13 [34.2]	23 [37.7]	4 [22.2]	0.518
Follow-up [months], median IQR	102 [76–141]	107 [85–140]	102 [72–149]	102 [70–145]	0.813
Prophylactic postoperative therapy					
Steroids	15 [12.8%]	4 [10.5]	9 [14.8]	2 [11.1]	0.928
Thiopurines	20 [17.1]	7 [18.4]	7 [11.5]	6 [33.3]	0.108
Biologics	11 [9.4]	4 [10.5]	4 [6.6]	3 [16.7]	0.352
Active inflammation distal resection margin	18/74 [24.3]	6 [26.1]	7 [18.4]	5 [38.5]	0.341

IQR, interquartile range.

An inflamed appendix was observed in 38/99 patients [39.4%], and in 61 patients the appendix showed no signs of inflammation. No significant differences in patients and prognostic relevant disease characteristics were found between these two groups. Disease duration at time of ICR was 41 months [8–72] in the inflamed group and 49 months [18–127] in the group with no signs of inflammation in the appendix. Patients with an inflamed appendix were slightly younger. Ileocolic Crohn’s disease was reported in 15/38 [40%] and 22/61 [36%], respectively. Furthermore, active inflammation in the colonic resection margin was not associated with inflamed appendices, with 26% positive margins in the inflamed group and 18% in patients with no signs of inflammation in the appendix. Penetrating disease was reported in 40% vs 31%, respectively. Inflammatory disease was predominantly seen in patients with inflamed appendices [37 vs 20%], and stricturing disease was more common in patients with no signs of inflammation in the appendix [49 vs 24%], Table 1.

In contrast, patients with previous appendectomy had a shorter disease duration at time of ICR when compared with those with and without an inflamed appendix: 37 months vs 46 months, Table 2. Numerically, patients with previous appendectomy were more often on postoperative thiopurines and biologics compared with the other groups. There was no difference in disease location [L1 or L3], but penetrating disease was significantly more often seen in the group with previous appendectomy: 56% vs 34%, *p* = 0.038 [Table 2].

**Table 2:** Baseline and disease characteristics, no appendectomy vs previous appendectomy

Baseline characteristics, *n* [%]	No appendectomy [*n *= 99]	Previous appendectomy [*n* = 18]	*p*-value
Gender, female	68 [68.7]	9 [50]	0.176
Age at surgery [years], median IQR	30 [24–41]	35 [25–47]	0.278
Duration of disease [months], median IQR	46 [15–122]	37 [8–79]	0.387
Smoking, active	33 [33.3]	1 [5.6]	0.021
Age at diagnosis			0.914
Montreal A1	11 [11.1]	2 [11.1]	
Montreal A2	74 [74.7]	13 [72.2]	
Montreal A3	14 [14.1]	3 [16.7]	
Location of disease			0.605
Montreal L1	62 [62.6]	10 [55.6]	
Montreal L3	37 [37.4]	8 [44.4]	
Behaviour of disease at surgery			0.255
Montreal B1	26 [26.3]	3 [16.7]	0.557
Montreal B2	39 [39.4]	5 [27.8]	0.189
Montreal B3	34 [34.3]	10 [55.6]	0.038
Perianal disease	21 [21.2]	3 [16.7]	1.000
Preoperative therapy <12 weeks			0.151
Steroids	42 [42.4]	7 [38.9]	
Thioprines	38 [38.4]	7 [38.9]	
Biologics	36[26.4]	4 [22.2]	
Follow-up [months], median IQR	103 [76–141]	102 [70–145]	0.967
Prophylactic postoperative therapy			
Steroids	13 [13.1]	2 [11.1]	1.000
Thioprines	14 [14.1]	6 [33.3]	0.081
Biologics	8 [8.1]	3 [16.7]	0.372

IQR, interquartile range.

### 3.1. Recurrences

**Figure 2:**
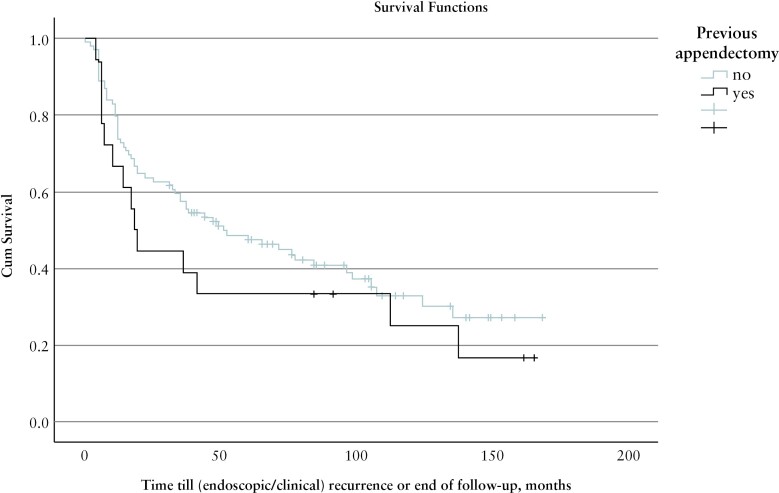
Kaplan–Meier analysis: no appendectomy vs previous appendectomy.

Median follow-up time was 102 months [76–141]. In total, disease recurrence [endoscopic and/or clinical] was reported in 76/117 patients [65%]: 62% in the inflamed appendix group, 63% in patients with no signs of inflammation in the appendix, and 78% in the previous appendectomy group. The Kaplan–Meier analysis comparing recurrence-free survival rate in the previous appendectomy group and the groups with and without an inflamed appendix at time of ICR, showed a trend towards a higher recurrence rate, although not statistically significant [*p* = 0.270], Figure 2.

There was no difference in intervention-related recurrence rates between the three groups. Intervention-related recurrence occurred in the total cohort in 23/117 patients [20%]: in 13% of patients with an inflamed appendix, 23% of patients with no signs of inflammation in the appendix, and 22% of patients with a previous appendectomy.

All analyses performed with the obliterated appendices included in the ‘inflamed appendix’ group did not affect any outcomes.

## 4. Discussion

The results of this study suggest that, although the appendix is anatomically part of the colon, an inflamed appendix itself should not be considered L3 disease. There appears to be no association between inflammation in the appendix at the time of ICR and disease location, disease severity, or prognosis. In contrast, a previous appendectomy shows a trend towards a worse phenotype, with increased numbers of penetrating disease and an increased recurrence rate compared with patients without previous appendectomy.

These initial findings are somewhat counterintuitive. A previous study showed that patients with active inflammation at the distal resection margin of the colon after ICR for CD had a different phenotype, which was associated with a significantly increased risk of recurrence after surgical resection. It was considered that active inflammation in the distal resection margin identifies undiagnosed L3 disease.^11^ In contrast to those results, the current data seem to suggest that inflammation of the appendix is not associated with disease location, despite its distal location. On the other hand, a study by Li *et al*. showed that history of appendectomy in CD was associated with a higher risk of progression of L1 to L3 disease at second surgical resection.^15^ This makes sense, as the appendix contains all colonic layers and is therefore a true diverticulum of the colon.^16^ This could be consistent with our initial hypothesis that because of the colonic location of the appendix, previous appendectomy involvement identifies an undiagnosed L3 disease. However, in the current study, no analysis could be performed on second surgical resection in the group who underwent appendectomy before ICR, as numbers were too small to perform meaningful analysis.

In contrast, previous appendectomy shows a worse disease course with an associated worse prognosis [more use of biologics and a trend towards more recurrences postoperatively] compared with those without previous appendectomy, which is in line with literature.^6^

Interestingly, appendix behaviour seems to be associated with CD behaviour rather than location, possibly indicating a different form of phenotypic characteristic. Inflammatory disease was predominantly seen in patients with inflamed appendices, stricturing disease was more common in patients with no signs of inflammation in the appendix, and penetrating disease was most frequently observed in patients with previous appendectomy. The latter might suggest that patients with previous appendectomy had early severe disease complications/transmural disease, which could also explain why a trend towards poorer prognosis is seen in this group in the current study and in previous literature.^6^ Due to small numbers in the appendectomy group, it is not possible to draw firm conclusions.

When looking at the current results, it seems that an affected appendix could be seen as a coincidental finding or a phenotypic characteristic [disease behaviour]. Obviously, an appendicitis-like histological finding in the resection specimen might just be seen as an innocent bystander effect. Any mass [colorectal carcinoma] or inflammatory process may cause acute appendicitis, either by way of direct obstruction of the appendiceal lumen or as a result of adjacent inflammation and oedema.^17–19^

Another interesting hypothesis is the involvement of the mesentery in CD. These days, we know that the mesentery is not just an innocent bystander, but might have an active role in CD. Fat wrapping, or creeping, fat, is the hallmark of terminal ileitis, and mesenteric contributions explain the topographical distribution of Crohn’s disease.^20–22^ In that context, it is interesting to realise that the meso-appendix is the portion of the mesentery connecting the ileum to the appendix. If the concept is correct that the mesentery drives the disease in the terminal ileum, it might as well drive inflammation in the appendix, given the location of the meso-appendix in relation to the mesentery of the terminal ileum.

Previous conflicting reports fuel the discussion as to whether appendicitis increases the risk of CD, or if people at risk for CD are more likely to develop appendicitis.^23^ We cannot comment on that finding, as the current results are not part of an epidemiological study with incidences of CD. However, 9.9% of patients included in this study underwent an appendectomy prior to their ICR, which is comparable to the lifetime prevalence of appendicitis in Western countries.^24,25^ On the other hand, this study only included patients with L1 and L3 disease. Patients with only colonic disease [L2] were not included. Whether the incidence of a previous appendectomy would increase when including L2 disease, cannot be deducted from these data. However, the fact that the prevalence is as high as in the Western population, suggests that this is a representative population.

This study has several limitations inherent in its retrospective nature. Although all microscopic aspects of pathology reports were reviewed by an experienced pathologist specialised in IBD, this does not make up for the variability in pathological reporting over the years. The appendix is not part of the standard pathological assessment of ICR resection specimens. In addition, the relatively small number of patients in this series from a single-centre cohort may limit generalisability, although the data come from a large tertiary referral centre with a large catchment area. A strength of the study is its prospective data collection with few missing data, which assures clinical accuracy.

In conclusion, the current study could not confirm a different prognosis for patients with and without an inflamed appendix in an ileocaecal resection specimen. Interestingly, although the appendix is anatomically part of the caecum, the incidence of an inflamed appendix was comparable in L1 and L3 disease. In contrast, in patients with a previous appendectomy, a trend was seen towards increased postoperative recurrence, which might be related to a higher incidence of penetrating disease.

## Data Availability

The data that support the findings of this study are available from EvdDW, upon reasonable request.
